# Research Updates on the Mechanism and Influencing Factors of the Photocatalytic Degradation of Perfluorooctanoic Acid (PFOA) in Water Environments

**DOI:** 10.3390/molecules28114489

**Published:** 2023-06-01

**Authors:** Jie Liang, Lingling Guo, Biao Xiang, Xueyi Wang, Jiaxi Tang, Yue Liu

**Affiliations:** 1 School of Environmental Science and Engineering, Liaoning Technical University, 47 Zhonghua Road, Xihe District, Fuxin 123000, China; liangjie2021202110@163.com (J.L.);; 2 Microbial Research Institute of Liaoning Province, Chaoyang 122000, China

**Keywords:** perfluorooctanoic acid, degradation, catalytic, influencing factors, mechanism

## Abstract

Perfluorooctanoic acid is ubiquitous in water bodies and is detrimental to the health of organisms. Effectively removing perfluorooctanoic acid (PFOA), a persistent organic pollutant, has been a hot topic around the world. With traditional physical, chemical, and biological methods, it is difficult to effectively and completely remove PFOA, the costs are high, and it is easy to cause secondary pollution. There are difficulties in applying some technologies. Therefore, more efficient and green degradation technologies have been sought. Photochemical degradation has been shown to be a low-cost, efficient, and sustainable technique for PFOA removal from water. Photocatalytic degradation technology offers great potential and prospects for the efficient degradation of PFOA. Most studies on PFOA have been conducted under ideal laboratory conditions at concentrations that are higher than those detected in real wastewater. This paper summarizes the research status of the photo-oxidative degradation of PFOA, and it summarizes the mechanism and kinetics of PFOA degradation in different systems, as well as the influence of key factors on the photo-oxidative degradation and defluoridation process, such as system pH, photocatalyst concentration, etc. PFOA photodegradation technology’s existing problems and future work directions are also presented. This review provides a useful reference for future research on PFOA pollution control technology.

## 1. Introduction

Perfluorooctanoic acid (PFOA) is a synthetic chemical in which fluorine atoms replace all the carbon-linked hydrogen atoms [1]. PFOA has good hydrophobic and oleo-phobic properties and high chemical stability and has been used in papermaking, leather, material packaging, textile, cosmetics, fire protection, and other industries [2,3,4]. Due to the high C–F bond energy (484 KJ/mol), it is not easily degraded under high temperatures, intense light, and certain biological conditions [5]. It has been detected in air, soil, water, plants, and even human serum all around the world [6,7,8]. In addition to their persistence in environmental media, PFASs are also harmful to human health, causing diseases of the thyroid, reproductive system, respiratory system, and kidney system [9,10]. In 2009, perfluorooctane sulfonate (PFOS) and its salts were officially included in the list of additional POPs at the fourth Conference of the Parties to the Stockholm Convention. In 2019, perfluorooctanoic acid (PFOA) and related compounds were added [11,12]. At present, many developed countries have restricted or eliminated the production and use of PFAS-based products, especially PFOS and PFOA. However, the production and use levels of PFASs in China have increased yearly, and China has become the primary producer and consumer of PFASs and related substances [13].

In recent decades, PFASs have been widely detected in the environment, and they are present in almost all water bodies, such as surface water, groundwater, etc. [14]. It is estimated that approximately 23–506t of PFOA is discharged into the atmosphere every year. In total, 1 to 13 tons of PFOA are deposited into terrestrial environments [15]; the water environment is the final destination of PFOA, and this may ultimately affect the safety of drinking water. It is reported that drinking water in many cities and regions of China has been contaminated by varying degrees of PFOA. In particular, a higher content of PFOA was detected in the cities distributed along the Yangtze River basin, e.g., Zigong (502.9 ng/L), Lianyungang (332.6 ng/L), and Changshu City (122.4 ng/L), among others [16]. It’s first suggested health advisory values of 85 ng/L for PFOA for China, in 2019 [17].

Due to the chemical structure of PFOA, PFOA shows high stability against chemical and thermal damage, and most degradation techniques cannot fundamentally damage it [18]. Currently, pollution control technologies for PFOA mainly include photocatalytic degradation, ultrasonic pyrolysis, adsorption treatment, electrochemical oxidation, and microbial degradation [19,20,21,22,23]. Ultrasonic thermal degradation technology is not mature enough, and most studies are limited to experimental conditions with unknown practical applications [19]. Adsorption treatment needs to consider the secondary recovery of the adsorbent. Electrochemical oxidation has a poor treatment effect on low concentrations, high energy consumption, and high costs [24]. The degradation speed of microbial treatment technology is not efficient [25].

Meanwhile, photocatalytic degradation has been associated with PFOA degradation due to its high efficiency, low cost, and environmentally friendly characteristics [26]. Studies have shown that catalysts or oxidants are usually added to improve degradation efficiency [27,28]. 

Therefore, this paper explores the indirect photodegradation mechanism of PFOA in different systems. Compared with other reviews, this paper summarizes a more comprehensive system and summarizes the main factors affecting PFOA photodegradation. Since the cost of photolysis treatment increases with increased reaction time, we added degradation kinetics and byproducts to the analysis, which has rarely been seen in previous studies, to provide a theoretical basis for improved degradation technology. Additionally, the paper presents the problems and challenges of PFOA photodegradation technology in practical applications. It is hoped that this article will provide readers with deep insights into the mechanism of photo-oxidative degradation of PFOA and contribute to the development of effective photocatalytic technologies.

## 2. Mechanisms of PFOA Photo-Oxidation Degradation

### 2.1. Direct Photodegradation Mechanisms

**Figure 1:**
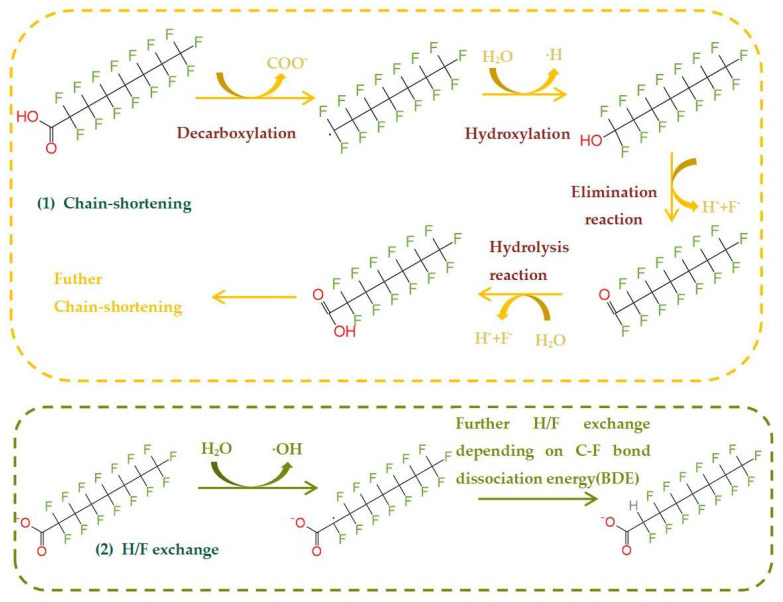
Direct photodegradation of the PFOA.

The absorption band of PFOA has an overlap with the UV absorption spectrum, and PFOA absorption is strong from the deep UV zone up to 220 nm and weak from 220 to 460 nm, which enables the direct photolysis of PFOA under UV light [29]. First (Figure 1), PFOA undergoes direct illumination, causing the breakage of C–C bonds between C_7_F_15_ and COOH (Equation (1)); then, the water detachment fluoride forms a short-chain perfluorinated compound (Equations (2)–(4)).

PFOA→C_7_F_15_· + ·COOH (1)

C_7_F_15_· + H_2_O→C_7_F_15_OH + H (2)

C_7_F_15_OH→C_6_F_13_COF+ H^+^ + F^−^ (3)

C_6_F_13_COF + H_2_O→C_6_F_13_COOH+ H^+^ + F^−^ (4)

### 2.2. Mechanism of Oxidative Photodegradation of PFOA

Given the visible light response, electronic band structure, and thermal and chemical stability [28], by adding oxidants (persulfate, periodate, carbonate, etc.), free radicals with strong oxidation properties (such as sulfate radicals, periodate radicals, and carbonate radicals) can be produced under ultraviolet irradiation to improve the degradation rate of PFOA [30].

Table 1 shows the reaction conditions and degradation efficiency levels of the PFOA oxidative photodegradation technique. The processes and mechanisms of PFOA degradation will be described in detail according to the degradation system.

The photodegradation mechanisms of PFOA in the different systems are analyzed below.

The PFOA degradation ratio is calculated using (Equation (5)):

(5) $$\begin{array}{l} d_{PFOA}=\frac{C_{0}-C_{t}}{C_{0}}\times 100\% \end{array}$$

Here, *d_PFOA_* is the degradation ratio of PFOA, *C*_0_ is the initial PFOA concentration before irradiation (μM), and *C_t_* is the PFOA concentration at time *t* (μM).

The defluorination ratio of PFOA is calculated using (Equation (6)):

(6) $$\begin{array}{l} {d_{F}}^{-}=\frac{{C_{F}}^{-}\times M_{W}PFOA}{C_{0}\times n\times M_{W}F}\times 100\% \end{array}$$

where *d_F_^−^* is the PFOA defluorination ratio, *C_F_*^−^ is the fluorine ion concentration at time *t* (μM), *C*_0_ is initial concentration of PFOA (μM), *M_w_PFOA* is the molecular mass of PFOA, *M_w_F* is molecular mass of fluorine, and *n* is the number of fluorine atoms in the PFOA molecule (*n* = 15).

Pseudo-first-order kinetics (Equation (7)):

(7) $$-\ln\frac{C_{t}}{C_{0}}=k_{1}t+b$$

where *C_t_* indicates the concentration of PFOA at some point after degradation, μg/L; *C*_0_ indicates the concentration of PFOA before degradation, μg/L; $\frac{C_{t}}{C_{0}}$ represents the degradation rate of the PFOA; *k*_1_ indicates the first-order kinetic reaction constants; *t* indicates the reaction time; and *b* is the constant.

Pseudo-second-order kinetics (Equation (8)):

(8) $$\frac{1}{C_{t}}-\frac{1}{C_{0}}=k_{2}t$$

where *C_t_* indicates the concentration of PFOA at some point after degradation, μg/L; *C*_0_ represents the concentration of PFOA before degradation, μg/L; *k*_2_ represents the second-order kinetic reaction constants; and *t* is the indicated reaction time.

#### 2.2.1. Degradation Mechanism in Persulfate Systems

**Figure 2:**
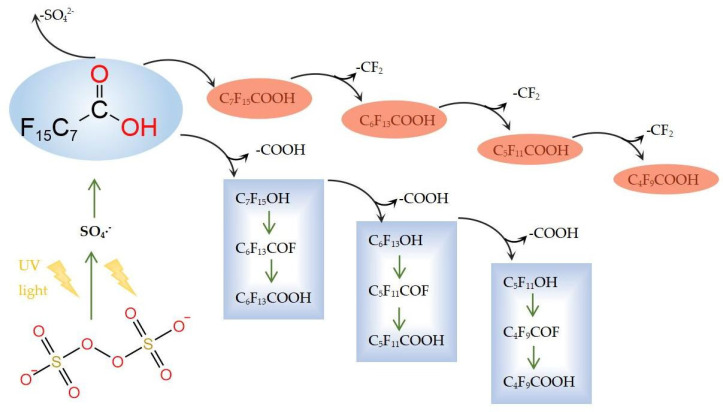
Photocatalytic degradation of PFOA using the persulfate system.

The degradation process of PFOA using persulfate can be explained using the results in Figure 2. During the decarboxylation process of carbon chain reduction, S_2_O_8_^2−^ is transformed into SO_4_·^−^ via illumination (Equation (9)), and the strongly oxidized SO_4_·^−^ takes one of the PFOA’s electrons to form C_7_F_15_COO· (Equation (10)). Carboxyl is electrically liberated to form ·C_7_F_15_ (Equation (11)). It then reacts with SO_4_^2−^ to form C_7_F_15_OSO_3_^−^ (Equation (12)); and then form C_7_F_15_OH (Equation (13)) [61,62]. The C_6_F_13_COOH is generated by sequential removal of the HF (Equations (14) and (15)).

The other stage refers to the gradual hydrogenation of and reduction in carboxylic acid where electrons are obtained directly by the PFOA at the cathode. Perfluorinated carboxylic acids are hydrogenated to form mono-hydrogen-substituted perfluorinated carboxylic acids, and then they are reduced to aldehydes and alcohols before being eliminated to generate alkenes. Finally, oxygen is added to generate mono-hydrogen or dihydrogen-substituted perfluorinated alkanes, as demonstrated in previous studies. In order to produce more in the system SO_4_·^−^, the Fe^2+^ is combined with UV light for persulfate activation purposes, which then increases the degradation rate of the PFOA.

S_2_O_8_^2−^ + *hv*→2SO_4_·^−^ (9)

C_7_F_15_COOH + SO_4_·^−^→C_7_F_15_COO· + SO_4_^2−^ (10)

C_7_F_15_COOH·→·C_7_F_15_ + CO_2_ + H^+^ (11)

·C_7_F_15_ + SO_4_^2−^→C_7_F_15_OSO_3_^−^ (12)

C_7_F_15_OSO_3_^−^ + H_2_O→C_7_F_15_OH + HSO_4_^−^ (13)

C_7_F_15_OH→HF + C_6_F_13_COF (14)

C_6_F_13_COF + H_2_O→HF + C_6_F_13_COOH (15)

#### 2.2.2. Degradation Mechanism in the TiO_2_ System

The band structures of some semiconductors offer photocatalytic activity and are often used as photocatalyst tools. The band structure is usually composed of a low valence band filled with electrons and an empty high-energy conduction band, located between the valence band and the conduction band. The energy difference is the band gap energy. When the catalyst is illuminated with sufficient power (greater than or equal to the band gap energy), the electrons in the valence band are excited and move from the forbidden band into the conduction band, generating holes at the position of the original electrons, as well as an electron–hole pair (e^−^-h^+^). Electron holes (h^+^) have strong oxidation; photobiology electronics (e^−^) can react to form reactive oxygen radicals, which can react with the organic matter adsorbed on the catalyst’s surface in order to mineralize its degradation.

We did not show any diffraction peaks on the XRD map of the untreated TiO_2_ nanoparticles, indicating that they have indefinite morphology. Their amorphous structure was also further demonstrated by high-resolution transmission electron microscopy testing of TiO_2_ [63].

**Figure 3:**
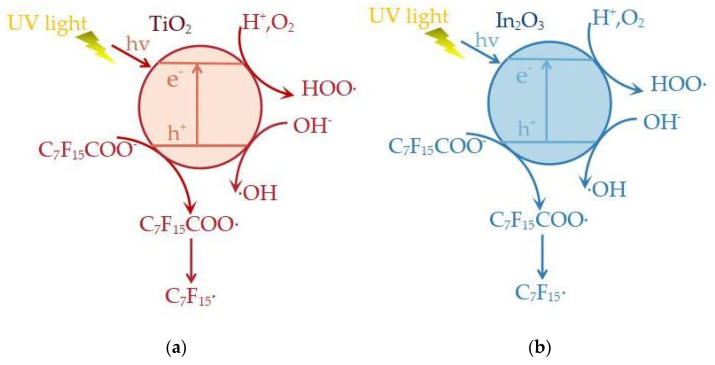
Photocatalytic degradation of PFOA using Ti O_2_ system (**a**) and In_2_O_3_ system (**b**).

In the PFOA/TiO_2_ system, PFOA is connected to its carboxylate group in the monodentate mode, allowing PFOA to form a tilted configuration on the TiO_2_ surface. The -CF_2_ group on the carbon chain of PFOA may interact with the OH group on the TiO_2_ surface through hydrogen bonds [48]. The degradation process is shown in (Figure 3a). Photogenerated holes and electrons (Equation (16)) are generated on the surface after TiO_2_ receives electrons, and the electron hole capture an e^−^ from the PFOA to form less stable C_7_F_15_COO· (Equation (17)), which decarboxylates to produce C_7_F_15_· and COO·, and C_7_F_15_· continues to react with ·OH to form C_7_F_15_COOH, which is defluorinated to form C_6_F_13_COF. C_6_F_13_COF is easily hydrolyzed into C_6_F_13_COOH. F^−^ is released into the water, and in this process, -CF_2_ is removed, and then short-chain perfluorinated carboxylic acids gradually remove -CF_2_ in the same way to achieve gradual degradation of intermediate products. Besides ·OH, the main active composite material also has h^+^ with strong oxidation ability, which can also participate in the reaction to remove -CF_2_ in the unit process. It can be seen (Equation (18)) that h^+^ is a key ion for PFOA degradation.

Since PFOA is inert to ·OH, the fluoride ions produced during degradation hinder the further action of TiO_2_, which makes pure TiO_2_ less effective at degrading PFOA [38]. The case of whether PFOA can be adsorbed on the surface of the catalyst in each system is crucial for the whole degradation process [64,65]. However, the degradation rate of PFOA can be significantly increased by the modification of TiO_2_. The modified mode is mostly concentrated in transition noble metallic-TiO_2_ transitions [40] and metal-TiO_2_ [41]. The modified TiO_2_ can effectively solve electron–hole binding and enhance the ability to react with water molecules to produce ·OH, thus improving the degradation rate of PFOA.

TiO_2_ + *hv* → TiO_2_(h^+^ + e^−^) (16)

C_7_F_15_COO^−^ + h^+^ → C_7_F_15_COO· (17)

h^+^ + HO^−^ → ·OH (18)

#### 2.2.3. Degradation Mechanism in the In_2_O_3_ System

By using scanning electron microscopy (SEM), In_2_O_3_ shows nanosheet-like structure, and lattice stripes can be clearly observed using high resolution transmission electron microscopy (HRTEM) [66].

Unlike the hole of TiO_2_, which converts to ·OH and then reacts with PFOA, the hole of In_2_O_3_ directly reacts with PFOA to form C_7_F_15_ radicals (Figure 3b). Photogenerated holes and electrons (Equation (19)) are generated on the surface after In_2_O_3_ receives electrons. Hydrolysis of h^+^ occurs to generate the ·OH (Equation (20)), C_7_F_15_COO^−^ and h^+^ to generate C_7_F_15_COO· (Equation (17)). The residual degradation pathway is similar to that of TiO_2_. The bidentate or bridging mode between the PFOA and In_2_O_3_ facilitates the transfer of electrons from the PFOA to the hole of In_2_O_3_. For In_2_O_3_ with different nanostructures, the unique porous structure gives it a larger surface area and a higher degradation rate of PFOA under the same mild conditions. Graphene-modified In_2_O_3_ increases the degradation rate of PFOA by increasing the reaction sites [50].

In_2_O_3_ + *hv* → In_2_O_3_(h^+^ + e^−^) (19)

h^+^ + H_2_O → H^+^ + ·OH (20)

#### 2.2.4. Degradation Mechanism in the Fe^3+^ System

**Figure 4:**
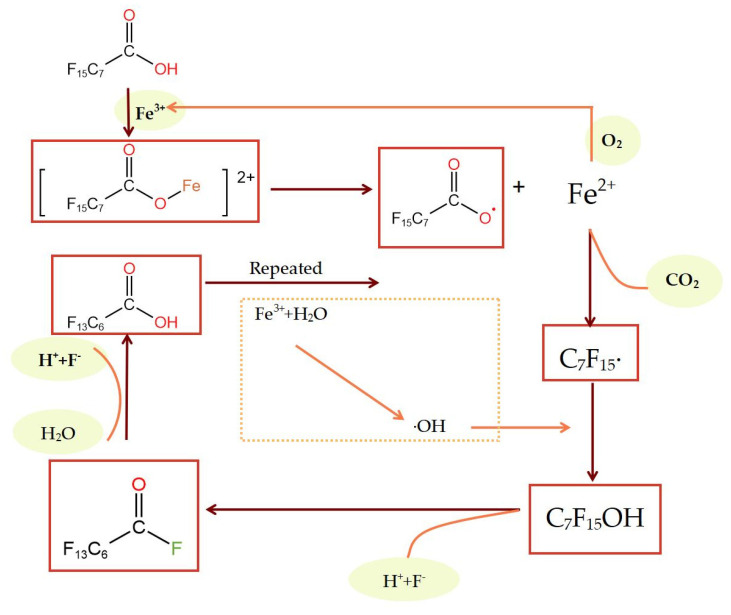
Photocatalytic degradation of PFOA using the Fe^3+^ system.

Fe^3+^ is easy to use to generate complex carboxylic acids with obvious photochemical characteristics, which undergo electron migration via ultraviolet radiation, making organic objects undergo oxidative degradation [67]. Fe^3+^ (Figure 4) will first form a complex with the PFOA [C_7_F_15_COO-Fe]^2+^ (Equation (21)) and then decompose into Fe^2+^ and C_7_F_15_COO· (Equation (22)) under UV light excitation. Afterwards, a hydrolysis reaction will generate C_6_F_13_COOH, HCOOH, and F^−^ (Equation (23)). Among them, Fe^2+^ forms after the PFOA is easily oxidized with Fe^3+^ using O_2_ in the air. Fe^3+^ can continue to be oxidized. However, Fe^3+^ is unstable and prone to precipitation under neutral or alkaline conditions, so the degradation efficiency of PFOA in the Fe^3+^ system is greatly affected by pH value [68,69,70,71,72].

C_7_F_15_COO^−^ + Fe^3+^ → [C_7_F_15_COO-Fe]^2+^ (21)

[C_7_F_15_COO-Fe]^2+^ + *hv* → Fe^2+^ + C_7_F_15_COO· (22)

C_7_F_15_COO· + 3H_2_O → C_6_F_13_COOH + HCOOH + 2F^−^ + 2H^+^ + ·OH (23)

#### 2.2.5. Degradation Mechanism in the H_2_O_2_/O_3_ System

When oxidants such as H_2_O_2_ and O_3_ are irradiated by UV light and absorb enough energy, non-selective OH radicals with strong oxidation properties are generated. PFOA generates ·C_7_F_15_ and ·COOH (Equation (24)) under illumination; ·C_7_F_15_ reacts with ·OH to form unstable C_7_F_15_OH (Equation (25)). C_7_F_15_OH generates C_6_F_13_COF (Equation (26)). C_6_F_13_COF hydrolysis generates C_6_F_13_COOH (Equation (27)). Based on Equations (25) and (26), the reaction is achieved following the detachment from a unit of HF; the reactant C_7_F_15_COOH to product C_6_F_13_COOH, due to the removal of the -CF_2_ group. C_6_F_13_COOH undergoes the same degradation pathway, and -CF_2_ gradually removes the degradation until mineralization is complete, and it finally forms carbon dioxide [73]. The process of removing the -CF_2_ group is also named the “flake off” [74].

C_7_F_15_COOH + *hv*→·C_7_F_15_ + ·COOH (24)

·C_7_F_15_ + ·OH→C_7_F_15_OH (25)

C_7_F_15_OH→C_6_F_13_COF + HF (26)

C_6_F_13_COF + H_2_O→C_6_F_13_COOH + HF (27)

#### 2.2.6. Degradation Mechanism in the NaIO_4_ System

When the system is NaIO_4_ (Table 1), the photolysis of IO_4_^−^ forms oxidized free radicals, such as IO_3_· and ·OH (Equation (28)). IO_3_· reacts with PFOA to generate [C_7_F_14_COOH]^+^· (Equation (29)) or generate C_7_F_14_COOH (Equation (30)). Then, [C_7_F_14_COOH]^+^·/C_7_F_15_COOH and ·OH reactions generate unstable C_6_F_13_OH (Equation (31)). C_6_F_13_OH generates C5F12COF (Equation (32)).

IO_4_^−^ + *hv* → IO_3_· + ·OH (28)

IO_3_· + C_7_F_15_COOH → [C_7_F_14_COOH]^+^· + F^−^ (29)

IO_3_· + C_7_F_15_COOH → C_7_F_14_COOH· + F^−^ (30)

[C_7_F_14_COOH]^+^·/C_7_F_14_COOH·+ ·OH → C_6_F_13_OH (31)

C_6_F_13_OH → C_5_F_12_COF + HF (32)

#### 2.2.7. Degradation Mechanism in the H_3_PW_12_O_40_ System

**Figure 5:**
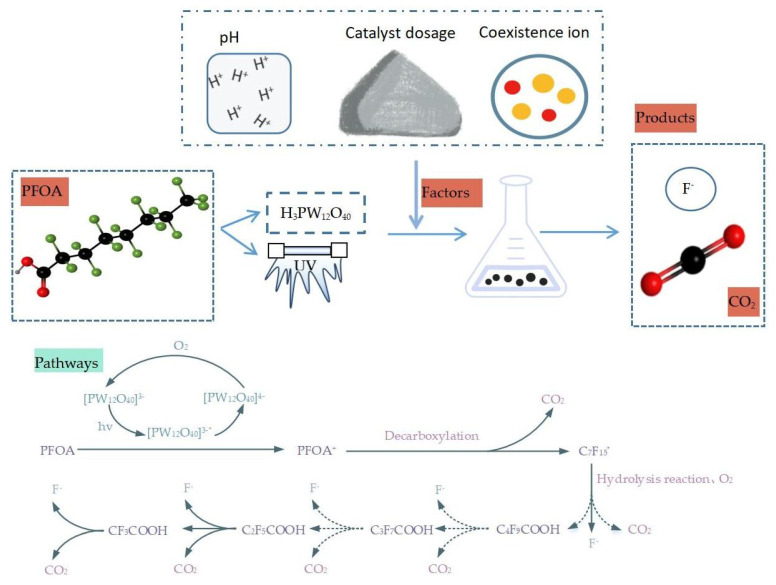
Photocatalytic degradation of PFOA using H_3_PW_12_O_40_ system.

Heteropolacid is a wide band material, which is prone to separate [75,76] from electron holes under ultraviolet radiation, and it can degrade some persistent organic matter [77]. In the H_3_PW_12_O_40_ system (Figure 5), efficient degradation of both CF_3_COOH and C_2_F_5_COOH can be achieved; the final degradation produces fluoride ions and carbon dioxide [75,78]. In this process, [PW_12_O_40_]^3−^ converts to an excited-state species via UV irradiation [PW_12_O_40_]^3−^* (Equation (33)). It takes one electron of the PFOA and is reduced to [PW_12_O_40_]^4−^ (Equation (34)). O_2_ can regenerate [PW_12_O_40_]^3−^, allowing the entire reaction cycle to proceed (Equation (35)). Subsequently, a decarboxylation reaction of the PFOA^+^ occurs to generate ·C_7_F_15_ (Equation (36)), followed by a hydrolysis reaction to generate C_4_F_9_COOH (Equation (37))[30,79].

[PW_12_O_40_]^3−^ + *hv* → [PW_12_O_40_]^3−^* (33)

[PW_12_O_40_]^3−^* + PFOA → [PW_12_O_40_]^4−^ + PFOA^+^ (34)

[PW_12_O_40_]^4−^ + O_2_ → [PW_12_O_40_]^3−^ + O^2−^ (35)

PFOA^+^ → ·C_7_F_15_ + CO_2_ (36)

·C_7_F_15_ + O_2_ + H_2_O → CO_2_ + F^−^ + C_4_F_9_COOH (37)

From the above system, although different oxidized radicals are produced in each system, it is clearly demonstrated that a common intermediate C_7_F_15_COO^−^ is produced during the degradation of PFOA. Due to the presence of the carboxylic acid group, the active substance will first attack the C–C bond between ·C_7_F_15_ and ·COOH, generating C_7_F_15_· and CO_2_. C_7_F_15_· will hydrolyze or react with ·OH to form an unstable C_7_F_15_OH; C_7_F_15_OH then undergoes an elimination reaction to generate C_6_F_13_COF, and it is then subjected to hydrolysis to generate C_6_F_13_COOH. C_6_F_13_COOH repeats the same degradation process, and the degradation of the PFOA is completed by gradually removing the –CF_2_ group. Finally, complete mineralization is realized. As different systems will produce different active substances, the degradation of PFOA will therefore vary. The photocatalytic degradation mechanism of PFOA can be divided into three groups according to the functional groups involved in the reaction:

(1) In the decarboxylation-hydroxylation-elimination-hydrolysis (DHEH) mechanism, the h^+^ or ·SO_4_^−^ radicals first form the carboxyl group, then seize carboxyl, form C_7_F_15_COO^−^, initiate spontaneous Kolbe decarboxylation, and convert into C_7_F_15_· and CO_2_. C_7_F_15_ reacts with H_2_O or ·OH to form C_7_F_15_OH. C_7_F_15_OH eliminates HF to form C_7_F_15_COF. C_7_F_15_COF is easily hydrolyzed and then removes HF and forms C_6_F_13_COOH. The C_6_F_13_COOH cycle mentioned in the above steps works by removing CO_2_ and HF in order to remove -CF_2_ [80].

(2) In the reductive decarboxylation mechanism, the photocatalyst surface produces photogenerated electrons; they first attack an α-carbon to generate unstable C_7_F_15_COO^2−^·, then C_7_F_15_COO^2−^· reacts with H^+^ to form C_7_F_15_· and HCOO^−^, and the subsequent reaction is consistent with the DHEH mechanism [81].

(3) ·O_2_^−^ and ·OH directly attack the C–C bond to convert C_7_F_15_COO^−^ into C_7_F_15_ with the DHEH mechanism [82].

## 3. Byproducts

During the degradation of PFOA, numerous intermediates are produced. Attention should be paid to the intermediates with toxicity. According to the literature, during the degradation of PFOA, the intermediates are mainly short-chain perfluorocarboxylic acids containing 2–6 carbon atoms. C_6_F_13_COOH, C_5_F_11_COOH, C_4_F_9_COOH, C_3_F_7_COOH, and C_2_F_5_COOH were the most observed in experimental studies [83,84,85]. During the degradation process, the concentration of the above intermediates tends to first increase and then decrease [82]. The accumulation of short-chain perfluorine gradually increases, and the accumulation is positively correlated with the length of short chain perfluorine, indicating that the degradation is broken by the carbon chain, which is consistent with the degradation process reported in the literature [86].

Among them, the perfluorinated compounds containing 3–8 carbons can cause damage to the human respiratory tract. The longer the carbon chain, the more toxic it is to the organism [73].

The content of F- in the system is directly proportional to irradiation time. According to Table 1, the defluorination rate is lower than the degradation rate, which indicates that there are other fluoride compounds in the degradation process [87]. The total F content in the aqueous solution comprises the remaining PFOA, short-chain PFCAs, F^−^, and PFCAs, maintained at around 88%. Another 12% of the F content can be converted into gas-phase products [73].

In actual treatment, an understanding of how to reduce the toxicity of intermediates and further transform these substances until complete mineralization occurs remains to be studied.

## 4. The Main Factors Affecting the Photocatalytic Degradation of PFOA

The degradation of PFOA is influenced by solution pH, catalyst dosage, concentrations of PFOA, and coexistence ion, et al.

### 4.1. The pH in the Solution

In the photocatalytic degradation system of PFOA, the optimal pH value is related to the properties of the catalyst and PFOA [27]. It has been found that the degradation efficiency of PFOA under acidic conditions is better in the TiO_2_ system [40,88,89]. In the compound catalyst, the TiO_2_ surface is positively charged, and PFOA exists in the form of an anion, is adsorbed onto the surface of the catalyst material, and is oxidized by the active group on the surface of the material, thus promoting its degradation [56].

The pH value of the solution affects the degradation rate by affecting the distribution of the oxidant, the state and conversion of the free radicals, and the quantum yield of the oxidant [85]. The different results indicate that the optimal pH value for the other degradation conditions is in the acid range because the free radical yield is highest under these conditions [36,90]. The change in the hydrogen ion concentration in the reaction process may cause changes in active species and thus affect degradation. Under the constant conditions of the initial PFOA concentration and catalyst dosage, the degradation rate of PFOA decreases with an increase in pH value. Under the continuous needs of the initial PFOA concentration and catalyst dosage, the degradation rate of PFOA decreases with an increase in pH value [91]. A low pH may enhance free radical–radical interactions rather than free radical–pollutant interactions [92]. For example, Bentuo Xu [93] studied the degradation rate of PFOA (pH = 3, 5, 7, and 10) under different initial solution pH values, and the results show that the photodegradation rate of PFOA decreases from nearly 100% to 27%. This is because when the high pH oxidizes the holes to produce excess hydroxyl radicals, it inhibits PFOA degradation [45]. Generally speaking, pH has an important impact on the degradation of target pollutants in the persulfate system. When the pH of the system is alkaline, SO_4_^−^· will react with OH^−^ in the system to form SO_4_^2−^. However, at a lower pH, the degradation rate increases. This is likely due to the sulfate associated with the generated hydroxyl radical. However, in the Fe^3+^ system, precipitation occurs easily under neutral or alkaline conditions by transforming the electrons in the system. In addition, pH can also affect the dissociation of ionizable chemicals such as PFOA in solution, which in turn affects the photocatalytic properties. PFOA in the aqueous solution is mainly affected by C_7_F_15_COOH at pH < 2.8 and C_7_F_15_COO^−^ at pH > 2.8 [93]. At pH 2 and 3, after 3 h of light, the degradation rates of PFOA are 94.4% and 61.3%, respectively; when the pH of the reaction system increases to 4, 5, and 6, the degradation rates after 24 h of illumination are only 41.5%, 26.9%, and 20.7%, respectively. After 24 h of light at the pH of 2, 3, 4, 5, and 6, the defluorination rates are 38.6%, 34.1%, 23.8%, 18.1%, and 15.3% [27], respectively, and this changing pattern is consistent with the degradation rate. However, the defluorination rate is significantly lower than the degradation rate due to the C–F bond energy (485 kJ /mol) that is more significant than the C–C bond energy (332 kJ/mol).

### 4.2. Catalyst Dosage

In general, the degradation rate of PFOA increases with an increase in the transfer rate of electrons in the catalyst within a certain range. As the catalyst concentration increases, the reaction sites also increase, thus promoting the degradation rate. However, a high concentration of the catalyst will make the solution cloudy, reduce the transmittance of ultraviolet light, and reduce the degradation rate. Furthermore, with the continuous increase in catalyst concentration, the degradation rate only slightly increases and even decreases. At 0.5, 1.0, 1.5, and 2.0 g·L^−1^, the quasi-primary reaction kinetic equation is fitted with rate constants of 0.356, 0.522, 0.297, and 0.246 h^−1^ [27]. The degradation rate of PFOA increases with an increase in the S_2_O_8_^2−^ dose, especially at low dose levels, and the defluorination rate also increases with an increase in the dose of persulfate [94]. When the S_2_O_8_^2−^concentration exceeds 26.8 mM, the degradation rate of PFOA reaches a maximum and subsequently begins to decrease due to the saturation of SO_4_·^−^ concentration. The results show that when the concentration of Ti (IV)-doped Bi_2_O_3_ (BTO) is 0.5 g·L^−1^, BTO has the best PFOA removal efficiency (81.9%). When the catalyst concentration is higher than 0.5 g·L^−1^, solid particles block the propagation of light in the reaction system and increase the scattering of ultraviolet light. A strong light-shielding effect is generated, and the penetration thickness is reduced, resulting in a decrease in the utilization rate of light energy and the degradation effect of the catalyst [95]. When the concentration of Pd-TiO_2_ is 1 g·L^−1^, the degradation rate of PFOA reaches the maximum, but when the concentration of Pd-TiO_2_ continues to increase, the fluoride concentration actually decreases [96]. When the Pd-TiO_2_ increases, the amount of H^+^ and ·OH produced in the system also increases, which then increases the ratio of the concentration of active species to the concentration of PFOA; thus, the defluorination rate of PFOA is accelerated. However, when the concentration of Pd-TiO_2_ is too high, the particles gather together, reducing the production of surfactant and increasing the ultraviolet light scattering, leading to a reduction in the amount direct light in the solution and affecting the photocatalytic degradation rate. Therefore, the degradation of PFOA can be significantly promoted by adding an appropriate amount of catalyst. When added in excess, self-quenching can occur, hindering the degradation of PFOA.

### 4.3. Concentrations of PFOA

The degradation rate of PFOA increases with the electron transfer rate of the catalyst and PFOA, especially at low dose levels. The defluorination rate also increases with an increase in the dose of PFOA [97]. A PFOA concentration of 4 mM produces the highest PFOA degradation in the TiO_2_ system [61]. At a reaction temperature of 25 °C, without adjusting the initial pH reaction atmosphere in the air, the charge transfer rate of PFOA decreases with an increase in its concentration after 4 h of reaction [38]. At 3 h, the degradation rates of 1, 4, 7, and 10 mg·L^−1^ of PFOA are 98.1%, 94.4%, 80.4%, 80.3%, and 68.2%, respectively [27]. From the principle of photochemistry, under the condition of constant power of a UV lamp, the light density is constant. When the concentration of PFOA is low, the number of photons is large enough to react with PFOA and ensure a defluorination rate. When the concentration of PFOA is high, there are too many PFOA reaction points, and the number of generated photons cannot meet the charge transfer when generating excessive PFOA, leading to an increase in the defluorination rate [98].

### 4.4. Coexistence Ion

In the actual water environment system, multiple ions will coexist in the environment, and it will affect the degradation rate, so it is of great significance to study their impact on the degradation rate for actual water treatment. For example, PFOA usually coexists with mechanical pollutants, natural organic matter, bicarbonate, etc. Some can promote final degradation, while others do the opposite. Bicarbonate and organic matter can largely inhibit the decomposition of PFOA in wastewater. The competitive adsorption of UV light via bicarbonates and photocatalysts is important in order to decrease the degradation rate. Still, the effect of inhibition can be eliminated by regulating the pH value and adding ozone [48]. For the persulfate system, with nitrate and isopropanol (·OH capture agent), the degradation effect of PFOA significantly improves (91%), which may be attributed to a large number of ·NO_2_ radicals in the system, which greatly increases the removal rate of PFOA. In NaHCO_3_, the degradation effect of PFOA is greatly inhibited, due to the quenching of HCO_3_^−^ [58,99]. In real water, there will be other perfluorinated compounds, and the degradation rate of PFOA needs further study.

## 5. Kinetic Parameter

The degradation rate and defluorination rate depend on the target pollutant and the reaction conditions, such as the initial concentration of the target pollutant, solution pH, and catalyst concentration. The degradation of PFOA fits with a pseudo-first-order kinetic model, ranging from 7.5 × 10^−3^ to 1.44 min^−1^. Due to the presence of the C–F bond, the reaction time varies between 2 and 12 h. Different studies show that the defluorination rate is much lower than the degradation rate, indicating that PFOA is not fully converted to fluoride ions after the final degradation [73]. With the high degradation and defluorination rates in the UV/S_2_O_8_^2−^ system, the reaction rate constant ranges from 0.81 to 1.44 min^−1^ [37]. The CO_3_·^−^ process induced by HCO_3_^−^ during UV/H_2_O_2_ can improve the degradation of PFOA, with weak alkaline conditions facilitating the degradation of PFOA under UV and HCO_3_^−^ binding [60]. For PFOA, the defluorination rate is essentially lower than the degradation rate, indicating the formation of byproducts. Since the defluorination rate equals the mineralization rate, when the degradation rate is constant, the defluoridation rate is positively correlated with the degradation effect of PFOA.

Yao et al. selected a removal rate of PFOA between 20 and 120 min to calculate degradation kinetics and the process of pseudo-first-order kinetics and mesoporous TiO_2_ films of 6.3 × 10^−3^ min^−1^ and 3%-Sb_2_O_3_/TiO_2_ 12.6 × 10^−3^ min^−1^ [46]. It was found that with the addition of 3.6 mmol/L of the PFOA, the degradation rate constantly increases from 0.48/h to 0.88/h [30,87]. This is because zero-valent iron transforms Fe^2+^ to activate persulfate and promote the formation of SO_4_·^−^.

To better compare these photodegradation systems, the degradation kinetics of PFOA are summarized in Table 2.

## 6. Conclusions and Prospect

By studying the photocatalytic degradation of PFOA in various locations, we summarized the degradation pathway of PFOA in different systems and the factors affecting degradation rate. Meanwhile, deficiencies in studies of the degradation of PFOA were also found during the summary process. First, understanding degradation mechanisms and degradation intermediates is not comprehensive, and incomplete mineralized compounds can not be ignored; second, in the existing studies, the synergy of multiple degradation technologies is lacking; and lastly, current PFOA degradation studies are performed under laboratory conditions, and thus an actual water environment is lacking. To achieve rapid and efficient photocatalytic degradation, corresponding methods were adopted in different scopes. Future research directions will also focus on the following aspects:

(1) There is a need to enhance photocatalytic fluoride reduction and improve the defluorination efficiency of optimization strategies, while strengthening the type and toxicity of degradation intermediates. Further improvements to the catalyst are also required, such as noble metal surface deposition, metal ion and non-metal ion doping, etc.

(2) While degrading PFOA, the byproducts generated may threaten the environment. Finding ways to completely remove byproducts in the photocatalytic degradation process of PFOA is also a topic in current research. Further, studies will focus on the advantages of cooperative processing technology in PFOA degradation, improving the utilization rate of catalytic materials and reducing processing costs.

(3) While actively exploring green and efficient degradation technology of PFOA, alternatives to PFOA should also be explored. Research on the catalytic degradation of PFAAs under natural environmental conditions will lay the foundation for the practical application of water PFOA pollution remediation technology.

## Figures and Tables

**Table 1:** Photo-oxidative technologies for PFOA degradation.

Photochemical Catalyst		Light Wavelength (nm)	Power (W)	Initial Concentration of PFOA (mmol/L)	Reaction Time/h	Degradation Ratio/Defluorination Ratio/%	Ref.
None		254	200	1.35	72	89.5/33	[31]
		185	15	6 × 10^−2^	2	61.7/17.1	[32]
		185	23	2.4 × 10^−2^	3	100/50	[33]
		185 + 254	20	2.4 × 10^−3^ 1.2 × 10^−3^ 1.2 × 10^−4^	4	92.5/25 98.6/- 94.7/-	[34]
Persulfate		254	200	1.35	4	100/12	[35]
		254 (184)	23	6 × 10^−2^	2	64.8/18.3 (87.4/18)	[36]
Fe^2+^-Persulfate		254	9	2 × 10^4^	5	93.9/63.6	[37]
TiO_2_		254	500	4	6	30/22	[38]
		310–400	75	1.5	24	-/47	[39]
Doped metal	Pb-TiO_2_	254	400	0.121	12	99.9/22.4	[40]
		365	125	0.145	7	94.2/25.9	[41]
	Pt-TiO_2_	365	125	0.145	7	100/34.8	[41]
	Cu-TiO_2_	254	400	0.121	12	91/19	[42]
	Ag-TiO_2_	365	125	0.145	7	57.7/8.1	[41]
Loaded carbon material	TiO_2_-MWCNT ^1^	365	300	0.0725	8	94.4/-	[43]
	rGO-TiO_2_^2^	200–600	150	0.24	12	93 ± 7/62	[44]
Build heterojunction	Fe/TNTs@AC ^3^	254	30	0.241	4	>90/62	[45]
	Sb_2_O_3_-TiO_2_	200–800	4	0.0241	2	81.7/-	[46]
	Ce/TiO_2_/g-C_3_N_4_	420–800	300	9.64 × 10^−3^	3	94.4/38.6	[27]
	Ti_3_C_2_/TiO_2_	254	4.5	0.02	16	>99.9/49.0	[47]
In_2_O_3_		254	23	0.1	4	83.1/33.7	[48]
In_2_O_3_ NpNSs ^4^		254	23	0.0725	3	100/71	[49]
In_2_O_3_-graphene		254	15	0.0725	3	87/60.9	[50]
Different nanostructure In_2_O_3_		254	15	0.0725	20 min (microsphere) 40 min (nanoplates) 2 h (nanocubes)	100/-	[51]
g-C_3_N_4_-In_2_O_3_		254	500	0.482	-	91 (1 h)/96 (3 h)	[52]
In_2_O_3_-GR ^5^		254	15	0.0723	3	100/60.9	[50]
0.86%CeO_2_/In_2_O_3_		254	500	0.241	1	100/53.3	[53]
MnO_x_-In_2_O_3_		Natural	500	0.1205	3	99.8/17.4	[54]
Fe(III)		Natural light	-	0.0483	28d	97.8/12.7	[55]
Fe(III)		185	12	0.048	48	98/100	[56]
		254	23	0.048	4	78.9/38.7	[57]
Fe^0^ NPs ^6^		254	-	0.24	25	45.8 ± 6.5%/-	[58]
Periodate		254	23	0.010	2	70/17	[59]
Phosphotungstic		254	200	1.35	24	99.9/30	[31]
Carbonate		254	400	0.12	12	100/82.3	[60]

^1^: TiO_2_-MWCNT: Composite TiO_2_ of multiwalled carbon nanotubes. ^2^: rGO-TiO_2_: Reduced GO compound TiO_2_. ^3^: Fe/TNTs@AC: Iron-modified titanate nanotubes and activated carbon. ^4^: In_2_O_3_ NpNSs: In_2_O_3_ nanoporous nanospheres. ^5^: In_2_O_3_-GR: In_2_O_3_ graphene composite material. ^6^: Fe^0^ NPs: zero-valent iron nanoparticles.

**Table 2:** Comparison of PFOA degradation kinetics in different photodegradation systems under UV irradiation.

Photocatalyst	Kinetic Model	Degradation Rate (Optimal)	Ref.
In_2_O_3_ nanosphere	Pseudo-second-order	0.0175 L/(mg·min)	[100]
TiO_2_-rGO	Pseudo-first-order	0.163 h^−1^	[44]
H_2_O_2_/NaHCO_3_	Pseudo-first-order	0.370 h^−1^	[60]
Fe(NO_3_)_3_	Pseudo-first-order	2.26 h^−1^	[101]

## Data Availability

Not applicable.
